# Impact of Dementia on Patients Admitted With Complicated Diverticular Disease: A U.S. Nationwide Analysis

**DOI:** 10.7759/cureus.94971

**Published:** 2025-10-20

**Authors:** Anudeep Surendranath, Anupam Gupta, Saurabh Singhal

**Affiliations:** 1 Neurology, CHI St. Vincent Hot Springs, Hot Springs, USA; 2 Surgery, Bangalore Medical College and Research Institute, Bangalore, IND; 3 Surgery, Kempegowda Institute of Medical Science, Bangalore, IND; 4 Neurology, Opelousas General Health System, Lafayette, USA

**Keywords:** dementia, diverticular abscess, diverticular bleeding, diverticulitis, mortality

## Abstract

Background

Emerging research has suggested a link between diverticular disease and dementia, potentially mediated by alterations in the gut microbiome and chronic systemic inflammation. This study aimed to evaluate in-hospital outcomes in patients admitted with complicated diverticular disease (CDD) and pre-existing dementia.

Methods

We used the 2021 National Inpatient Sample (NIS), which reflects 97% of the U.S. population. Patients admitted with CDD, defined as diverticulitis, abscess, perforation, or bleeding, were identified and stratified by the presence or absence of a prior dementia diagnosis. In-hospital mortality was compared between groups. Subgroup analysis evaluated the impact of specific complications (perforation/abscess vs. diverticulitis/bleeding) on mortality among patients with dementia. Multivariable logistic regression was used to adjust for potential confounders.

Results

Of 221,460 patients admitted with CDD, 9,160 (4.1%) had a comorbid dementia diagnosis. Patients with dementia were older (mean age 82.5 years), predominantly female (64.9%), and more likely to have multiple comorbidities. Overall, in-hospital mortality was higher in the dementia group (unadjusted OR 3.41; 95% CI: 2.38-4.90; p < 0.01), though this was not statistically significant after adjustment (aOR 1.45; 95% CI: 0.97-2.16; p = 0.07). Subgroup analysis revealed that dementia was significantly associated with increased mortality among patients with perforation or abscess (aOR 2.07; 95% CI: 1.07-4.04; p = 0.03), but not in those with diverticulitis or bleeding alone.

Conclusion

Pre-existing dementia was associated with higher unadjusted mortality, but it was not statistically significant after adjustment, except in patients with CDD who presented with perforation or abscess.

## Introduction

Protrusions with a colonic mucosa characterize diverticulosis due to weak points in the wall [1]. Diverticular disease in this paper refers to a false diverticulum of the large bowel, characterized by mucosal and submucosal protrusion through the colonic wall with an overlying serosal covering but lacking the muscularis propria. Diverticulosis is a common disorder in the West. The progressive age is known to have a higher incidence of diverticular disease [1,2]. The etiopathogenesis of diverticular disease is not clearly understood. Diverticular disease is characterized by the protrusion of the mucosa at the sites where blood vessels, the vasa recta, penetrate the circular muscle of the colon [3]. It is hypothesized that this may be due to abnormal colonic motility, genetic factors, or changes in the gut microbiome [4]. Complicated diverticular disease (CDD) can manifest in multiple ways, ranging from inflammation as diverticulitis, abscess formation, bleeding, or free perforation [5,6]. It can present as an obstruction due to stricture formation, fistulization to surrounding organs, etc. Dietary factors, physical activity, obesity, and lifestyle factors like smoking and alcohol have all been known to play a possible role in the progression of the disease, and there is an increasing trend of diverticular disease being seen in the younger age group population [7].

Dementia is a disorder characterized by a decrease in cognition [8]. Diagnosis of dementia is made with the help of the Diagnostic and Statistical Manual of Mental Disorders, which helps identify a decreasing level of function severe enough to affect the daily functioning of an individual [9]. There has been an increasing incidence of dementia worldwide. There are multiple etiological factors associated with dementia, such as advancing age, hypertension, diabetes, smoking, high body mass index, congestive heart failure, renal insufficiency, use of certain medications, vascular causes, alcohol, chronic trauma, and certain infections like the human immunodeficiency virus [10,11].

There is increasing evidence that the intestinal microbiome and chronic inflammation are associated with dementia. It is estimated that there are more than 1000 pathogens of bacteria, protozoa, fungi, and other microbial flora in the gastrointestinal (GI) tract. It is hypothesized that changes in the microbiome of the GI tract can lead to inflammation and the release of substances that can lead to a neurohumoral response, which could have a local effect on the colon, leading to mucosal outpouching, and a systemic effect on the nervous system, causing progressive deterioration of memory, thinking, comprehension, language, and behavior [12,13].

The purpose of the study is to evaluate patients with complicated diverticular disease with pre-existing dementia and their outcomes. Despite emerging evidence linking diverticular disease and dementia through shared inflammatory and microbiome-mediated pathways, population-level data on their clinical interaction remain limited. To our knowledge, this is one of the few studies to use a nationally representative dataset, the 2021 National Inpatient Sample (NIS), to evaluate real-world in-hospital outcomes in this high-risk population. Further analysis is done to identify which complicated diverticular disease can worsen mortality in patients with pre-existing dementia.

## Materials and methods

Study design and database description

This study used the 2021 National Inpatient Sample (NIS), a comprehensive all-payer database developed by the Healthcare Cost and Utilization Project (HCUP) under the Agency for Healthcare Research and Quality (AHRQ) [14]. The study period spanned from January 1, 2021, to December 31, 2021, using data from the 2021 National Inpatient Sample (NIS). The NIS compiles data from 48 participating states, representing 97% of the U.S. population and 96% of discharges from community hospitals, excluding rehabilitation and long-term acute care facilities. It is designed as a 20% stratified sample of hospital discharges, ensuring representativeness across factors such as hospital ownership, bed capacity, teaching status, and geographic distribution. The NIS provides discharge-level sampling weights (DISCWT), strata (NIS_STRATUM), and primary sampling units (HOSP_NIS) that must be applied to generate nationally representative estimates. In this study, all descriptive and regression analyses were conducted using Stat StataCorp. 2025. Stata Statistical Software: Release 18. College Station, TX: StataCorp LLC.to account for the complex survey design, and results are presented as unweighted sample counts and weighted national estimates. This weighting process allows extrapolation to the U.S. inpatient population while accounting for stratification and clustering. The large sample size strengthens statistical power, enabling the detection of differences in patient populations and outcomes and making it a valuable tool for healthcare research. Since the data is de-identified and publicly available, this study did not require Institutional Review Board (IRB) approval and informed consent.

Study population

The study included all patients admitted with a principal diagnosis of complicated diverticular disease, identified using the International Classification of Diseases-10 (ICD-10) codes in the Appendix.

In this study, complicated diverticular disease refers to diverticular disease of the large bowel with diverticulitis, bleeding, abscess, or perforation. Complications such as stricture formation, obstruction, and fistulization to adjacent organs were excluded from the study. Additionally, diverticular disease of the small bowel or any associated involvement of the small bowel and adjacent organs was also excluded from the study.

Dementia ICD-10 codes included Alzheimer's disease, vascular dementia, unspecified dementia, Pick’s disease, other frontotemporal neurocognitive disorders, and dementia with Lewy bodies.

The study population was divided into two groups based on the presence or absence of a history of dementia. We compared patient demographics and hospital characteristics between these groups to evaluate clinical presentation and outcome differences.

Outcomes

The primary outcome of interest in this study was in-hospital mortality, comparing patients with and without a diagnosis of dementia who were admitted with a principal diagnosis of complicated diverticular disease. Mortality was defined as death occurring during the index hospitalization.

In addition to evaluating overall mortality, a subgroup analysis was conducted to assess whether the association between dementia and in-hospital mortality differed based on the presence or absence of diverticular disease complications, specifically perforation or abscess. Patients were stratified into two subgroups: those with perforation or abscess and those with diverticulitis or bleeding. The effect of dementia on in-hospital mortality was then evaluated separately within each subgroup to explore potential effect modification by disease severity.

Multivariable logistic regression was used to estimate the odds of mortality in each group, adjusting for confounding variables including age, sex, race/ethnicity, insurance type, comorbidity burden (Charlson Comorbidity Index), and hospital-level factors such as region, teaching status, and urban location.

Statistical analysis

Data analysis was conducted using StataCorp. 2025. Stata Statistical Software: Release 18. College Station, TX: StataCorp LLC. For continuous variables, p-values were determined using t-tests, while Fisher’s exact test was employed for categorical and binary variables. To assess the relationship between a diagnosis of dementia and binary outcomes, multivariable logistic regression analysis was performed, whereas multivariable linear regression was utilized for continuous outcomes. Both models were adjusted for potential confounders to improve the precision and reliability of the findings. Additionally, subgroup analyses were conducted to explore potential effect modification by age (<75 vs. ≥75 years) and sex. The multivariable logistic regression model used to estimate the adjusted odds of in-hospital mortality included the following covariates: age, sex, race/ethnicity, insurance type, Charlson Comorbidity Index score, and hospital-level factors such as region, teaching status, and urban location. The regression equation takes the form: logit(p) = β₀ + β₁(Dementia) + β₂(Age) + β₃(Sex) + β₄(Race) + β₅(Insurance) + β₆(CCI) + β₇(Hospital Region) + β₈(Teaching Status) + β₉(Urban Location), where p represents the probability of in-hospital mortality. Statistical significance was defined as a two-sided p-value < 0.05.

## Results

Patient and hospital characteristics: 221,460 patients were identified in the 2021 National Inpatient Sample (NIS) with a principal diagnosis of complicated diverticular disease. Of the 221,460 patients admitted with complicated diverticular disease (CDD), 9,160 patients (4.14%) had a comorbid dementia diagnosis. Table 1 presents a detailed comparison of patient demographics and hospital characteristics between the dementia and non-dementia groups.

**Table 1:** Patient and hospital characteristics Legend: Values are presented as counts (weighted %) or means (95% CI). Continuous variables were compared using independent t-tests. Categorical variables were compared using Pearson’s χ² test, except where expected cell counts were < 5, in which case Fisher’s exact test was applied (notably for Native American race and self-pay insurance groups).

	No dementia	Dementia	Test Statistic	p-value
Female (%)	120,000 (54.83)	5945 (64.94)	χ² = 250.94	<0.01(1.62 × 10⁻⁵⁶)
Mean age (95% conf. interval)	63.48(63.30-63.66)	82.51(82.17-82.84)	t = -98.08	<0.01(0.000e+00)
Race/ethnicity (%)			χ² = 232.92	<0.01(2.536e-48)
White	150,000 (72.14)	6255 (70.04)		
Black	25,000 (12.14)	1500 (16.8)		
Hispanic	24,000 (11.52)	805 (9.01)		
Asian or Pacific Islander	3250 (1.52)	165 (1.85)		
Native American	935 (0.45)	15 (0.17)		
Other	4630 (2.23)	190 (2.13)		
Median annual income in patient’s zip code, US$, no. (%)			χ² = 35.40	0.05
$1 - $51,999	53,000 (25.27)	2555 (28.12)		
$52,000 - $65,999	52,000 (24.99)	2190 (24.11)		
$66,000 - $87,999	53,000 (25.51)	2195 (24.16)		
$88,000 or more​	51,000 (24.24)	2145 (23.61)		
Insurance type, no. (%)			χ² = 6581.70	< 0.0001
Medicare	100,000 (49.58)	8340 (92.56)		
Medicaid	20,000 (9.61)	150(1.66)		
Private insurance, including Health Maintenance Organizations (HMO)	76,000 (36.71)	480 (5.33)		
Self-pay	8470 (4.1)	40 (0.44)		
Hospital region, no. (%)			χ² = 48.41	1.74 × 10⁻¹⁰
Northeast	44,000 (20.68)	1775 (19.38)		
Midwest	48,000 (22.71)	1900 (20.74)		
South	83,000 (39.2)	3900 (42.58)		
West	37,000 (17.41)	1585 (17.3)		
Urban location (%)	190,000 (91.35)	8290 (90.5)	χ² = 9.38	0.002
Teaching hospital	150,000 (70.73)	6450 (70.41)	χ² = 0.23	0.629
Charlson Comorbidity Index score, no. (%)			χ² = 10605.99	<0.01
0	100,000 (47.02)	0 (0)		
1	47,000 (22.32)	2160 (23.58)		
2	26,000 (12.23)	2075 (22.65)		
≥3	39,000 (18.43)	4925 (53.77)		

Patients with dementia were significantly older, with a mean age of 82.51 years compared to 63.48 years in those without dementia (p < 0.01). Female patients comprised 5,945 of 9,160 patients with dementia (64.94%) versus 120,000 of 219,300 without dementia (54.83%) (p < 0.01).

Racial and ethnic distribution differed significantly (p < 0.01). The dementia group had a higher proportion of White patients (70.04% vs. 72.14%) and a lower proportion of Black patients (16.8% vs. 12.14%). Hispanic patients made up a smaller share of the dementia group (9.01% vs. 11.52%). The proportions of Asian or Pacific Islander, Native American, and other racial groups were comparable between the two groups.

Socioeconomic status, as estimated by median household income by ZIP code, showed marginal statistical significance (p = 0.05), with a slightly higher percentage of dementia patients in the lowest income bracket (<$52,000) compared to the non-dementia group (28.12% vs. 25.27%).

Insurance coverage showed notable differences (p < 0.01). Medicare was the predominant payer in the dementia group (92.56%) compared to 49.58% in the non-dementia group. Conversely, private insurance (5.33% vs. 36.71%), Medicaid (1.66% vs. 9.61%), and self-pay (0.44% vs. 4.1%) were less common in dementia patients.

Regional differences in hospitalizations (p = 0.04) were observed, with a slightly higher proportion of dementia patients admitted in the South (42.58% vs. 39.2%). However, the proportion of patients treated in urban hospitals (90.5% vs. 91.35%, p = 0.19) and teaching hospitals (70.41% vs. 70.73%, p = 0.77) was similar between the groups.

Comorbidity burden, assessed by the Charlson Comorbidity Index (CCI), was significantly higher in the dementia group (p < 0.01). Over half of dementia patients (53.77%) had a CCI score ≥3 compared to only 18.43% of non-dementia patients. Notably, none of the dementia patients had a CCI score of 0, while nearly half (47.02%) of the non-dementia group fell into this category.

Primary outcome

Among the 221,460 patients admitted with a principal diagnosis of complicated diverticular disease, 1340 (0.6%) died during hospitalization. As shown in Table 2, patients with a comorbid diagnosis of dementia exhibited a higher risk of in-hospital mortality compared to those without dementia. The unadjusted odds ratio (OR) for mortality in patients with dementia was 3.41 (95% CI: 2.38-4.90, p < 0.01), indicating a significantly increased unadjusted risk. However, after adjusting for potential confounders, including age, sex, race, insurance status, comorbidity burden, and hospital characteristics, the association was attenuated and no longer statistically significant, with an adjusted OR of 1.45 (95% CI: 0.97-2.16, p = 0.07).

**Table 2:** Unadjusted and adjusted odds ratios for in-hospital mortality in patients with complicated diverticular disease, stratified by dementia status Adjusted odds ratios were derived from a multivariable logistic regression model that controlled for age, sex, race/ethnicity, insurance type, Charlson Comorbidity Index score, and hospital-level characteristics, including region, teaching status, and urban versus rural location.

Outcome	Exposure	Odds Ratio (OR)	95% Confidence Interval (CI)	p-value
Mortality (Unadjusted)	dementia	3.41	2.38-4.90	<0.01
Mortality (Adjusted)	dementia	1.45	0.97-2.16	0.07

Subgroup analysis (Table 3) was conducted to further evaluate whether the presence of complications of diverticular disease modified the effect of dementia on mortality, a subgroup analysis was performed. A comparison was done on the complicated diverticular disease with diverticulitis and bleeding, with patients having abscesses or perforations with or without bleeding. In patients with perforation or abscess, with or without bleeding, dementia was significantly associated with higher in-hospital mortality, with an adjusted OR of 2.07 (95% CI: 1.07-4.04, p = 0.03). In contrast, among patients with complicated diverticular disease with diverticulitis with or without bleeding, the association between dementia and mortality was not statistically significant (adjusted OR 1.34, 95% CI: 0.8-2.26, p = 0.27). These findings suggest that the adverse impact of dementia on mortality is more pronounced in patients with complicated diverticular disease and in patients who develop abscesses or perforations with or without bleeding.

**Table 3:** Subgroup analysis of in-hospital mortality by complication type in patients with and without dementia Values represent adjusted odds ratios (aOR) derived from multivariable logistic regression models, adjusted for age, sex, race/ethnicity, insurance type, Charlson Comorbidity Index score, and hospital-level factors (region, teaching status, urban location).

Subgroup	Odds Ratio (OR)	95% Confidence Interval (CI)	p-value
CDD + Perforation/Abscess ± Bleeding	2.07	1.07 – 4.04	0.03
CDD + Diverticulitis ± Bleeding	1.34	0.80 – 2.26	0.27

Subgroup analysis results are presented in Table 4. Among patients aged ≥75 years, dementia was significantly associated with increased in-hospital mortality (adjusted OR 1.76, 95% CI: 1.12-2.75, p = 0.01), while no significant association was observed in those <75 years (adjusted OR 1.23, 95% CI: 0.85-1.78, p = 0.26). The interaction p-value for age was 0.045, indicating significant effect modification. Similarly, dementia was associated with higher mortality in female patients (adjusted OR 1.58, 95% CI: 1.01-2.49, p = 0.04) but not in males (adjusted OR 1.42, 95% CI: 0.92-2.18, p = 0.11), with an interaction p-value of 0.038.

**Table 4:** Subgroup analysis of in-hospital mortality by age and sex Values represent adjusted odds ratios (aOR) with 95% confidence intervals, derived from multivariable logistic regression models adjusted for age, sex, race/ethnicity, insurance type, Charlson Comorbidity Index score, and hospital-level factors (region, teaching status, and urban location). Interaction p-values indicate the statistical significance of effect modification by age group and sex.

Subgroup	Adjusted OR	95% CI	p-value
Age < 75	1.23	0.85–1.78	0.26
Age ≥ 75	1.76	1.12–2.75	0.01
Interaction p-value (Age)	-	-	0.045
Male	1.42	0.92–2.18	0.11
Female	1.58	1.01–2.49	0.04
Interaction p-value (Sex)	-	-	0.038

## Discussion

Increasing age is associated with the development of diverticular disease and dementia [15,16]. In our database of about 221,460 patients with complicated diverticular disease, only 9,160 (4.14%) patients had a pre-existing diagnosis of dementia. The mean age group of patients with CDD and dementia was 82.51 years, versus patients with CDD without dementia of 63.48 years, who were substantially younger. The female population was significantly more significant, with 64.94% with a p-value <0.01. A total of 70% of the patients were white, with a higher prevalence of comorbidity and low socioeconomic status (<$52000 median annual income). Patients had predominant Medicare coverage in 92.56% of the patients. The southern United States had a higher presentation (42.58%).

The etiology of coexisting diverticulitis and dementia is not well defined. The hypotheses of the gut microbiome and inflammation as etiologies of diverticular disease and dementia are being increasingly considered [17]. There has been an association between an increase in microorganisms in legs, *Bacteroides*, *Alistipes*, and *Odoribacter*, and a decrease in* Lachnospira*. This microbial dysbiosis leads to changes in gut-brain access. This could lead to the release of mediators, leading to increased oxidative stress, neural damage, and deposition of β-amyloid [18]. There has been a reported increased presence of certain bacterial species, such as *Actinobacteria* and *Proteobacteria* species, in patients with dementia [19]. Diverticulitis occurs when there is bacterial trapping and the development of inflammation. In an acute setting, it can present with bleeding, inflammation, and progression to an abscess and perforation [20]. In patients with acute diverticulitis, distinct changes in the microbiome have been found, which may lead to precipitation of acute diverticulitis in diverticular disease [21]. Complicated diverticulitis below the age of 50 years is more common in men, and complicated diverticulitis and dementia are seen more commonly in females above the age of 50 [22], which is also seen in our outcome.

Our results are similar to those of multiple studies wherein the patients with lower economic status are more likely to be females and present with complications of diverticular disease in emergency settings [23]. This pattern is likely due to dietary and lifestyle factors, access to healthcare, insurance, and socioeconomic factors [24].

Acute manifestations of diverticular disease usually need an emergency visit. Many situations require admission and management, ranging from conservative management with antibiotics to invasive management such as colonoscopy, angioembolization, possible drainage of an abscess, and possible surgery, depending on the presentation [25,26]. Advancing age, medical comorbidities, and medications that cause immunosuppression or blood thinners are known to cause more morbidity and mortality [25-27].

Patients with complicated diverticular disease with pre-existing dementia had a higher risk of in-hospital mortality with OR 3.41 (95% CI: 2.38-4.90, p < 0.01). However, when we adjusted for potential confounding such as age, sex, socioeconomic status, insurance status, and comorbidity burden, the association was no longer statistically significant at 1.45 (95% CI: 0.97-2.16, p = 0.07).

Patients with complicated diverticular diseases with pre-existing dementia not only have advanced age, poor socioeconomic status, and poor insurance factors but also present more commonly in emergent situations with multiple comorbidities and advanced stages, which substantially contribute to the mortality outcomes. Hence, dementia as an independent factor was not a statistically significant contributor to mortality in our study.

Subgroup analysis of patients with complicated diverticular disease with pre-existing dementia presenting with diverticulitis, bleeding, or both did not have significantly higher inpatient mortality (adjusted OR 1.34, 95% CI: 0.8-2.26, p = 0.27) when compared to patients with similar complicated diverticular disease presentation without dementia.

Patients with chronic diverticular diseases (CDD) who also have dementia and experience perforations or abscesses, whether or not they have bleeding, have a statistically higher in-hospital mortality rate. The adjusted odds ratio (OR) is 2.07 (95% CI: 1.07-4.04, p = 0.03). This finding aligns with data indicating that patients of advanced age from lower socioeconomic backgrounds who present to emergency care often have multiple comorbidities and more severe presentations, which contribute to a higher mortality rate [28].

Limitations

This study relies on data from the National Inpatient Sample (NIS), an administrative, discharge-level database that assumes accurate coding of diagnoses and procedures. Therefore, some degree of misclassification bias cannot be excluded. Although the NIS represents approximately 20% of all hospital discharges and uses stratified weighting to generate national estimates, it remains a sample rather than a complete census; certain nuanced patient- or hospital-level characteristics may not be fully captured. Additionally, the NIS lacks detailed clinical variables such as disease duration, medication use, laboratory values, and temporal associations, which limits mechanistic interpretation.

Furthermore, inherent differences between dementia and non-dementia cohorts, particularly in age, sex, comorbidities, and socioeconomic status, may contribute to residual confounding, even after multivariable adjustment. The retrospective, cross-sectional design restricts causal inference between dementia and in-hospital outcomes in complicated diverticular disease (CDD). Despite these limitations, the findings underscore the clinical importance of early risk stratification and comprehensive care for elderly patients with dementia presenting with CDD. Future prospective, longitudinal studies incorporating clinical, microbiome, and inflammatory data are warranted to confirm these associations and explore underlying mechanisms.

## Conclusions

Only 4.14% of patients with complicated diverticular disease have pre-existing dementia. Female sex, advancing age, medical comorbidity, socioeconomic status, race, emergency presentation, geographic location, healthcare insurance, and dementia contribute to outcomes of in-hospital mortality.

Pre-existing dementia by itself does not show a statistically significant correlation with inpatient mortality in complicated diverticular disease unless associated with the perforation or abscess.

## Appendices

**Table 5:** Supplementary table with ICD codes

Diagnosis	ICD 10 codes
Complicated diverticular disease	K5720, K5721, K5731, K5732, K5733
Diverticular disease with perforation, abscess with or without bleeding	K5720, K5721
Diverticulitis with or without bleeding	K5731, K5732, K5733
Dementia	G30x, F01x, F03x, G31.01, G31.09, G31.83
